# Editorial: Human movement understanding for intelligent robots and systems

**DOI:** 10.3389/frobt.2022.994167

**Published:** 2022-08-17

**Authors:** Taizo Yoshikawa, Emel Demircan, Philippe Fraisse, Tadej Petrič

**Affiliations:** ^1^ Honda R&D Co.Ltd., Wako, Japan; ^2^ California State University, Long Beach, CA, United States; ^3^ Université de Montpellier, Montpellier, France; ^4^ Jožef Stefan Institute, Ljubljana, Slovenia

**Keywords:** robotics, AI, biomechanics, kinesiology, intelligent

## 1 Human movement understanding

Robotics has become so sophisticated that the methods developed can be used to solve research questions in many other fields, from computer animation to neuroscience. In recent years, robotic computational strategies have contributed significantly to the analysis of human motion and manipulation capabilities. These analyses have led to advances in the field of robotics by enabling human-inspired capabilities in robots and simulated systems, as well as various learning methods for robots. They have also led to a deeper understanding of the human body and its motion generation strategies. Building on the methods and techniques developed in robotics, a variety of new, effective tools for synthesising human motion have also been introduced. These developments open new avenues for the study of human motion-with exciting prospects for clinical therapies, performance improvement, athletic training, and ergonomic assessment of workers. Extensive research has led not only to a deeper understanding of the human body and its motion generation strategy, but also to improvements in robotic technology that physically interacts with humans. There is a need for technologies that support people’s daily lives and technologies that physically support people’s movements. To this end, it is important to develop technologies in which a robot can estimate and predict a human’s movements and cooperate with the human’s movement intentions. While current robotics technologies rely on human models to mimic human-like behaviour in robots, they do so on a purely individual basis, while ignoring the behaviour and dynamics between partners that naturally occur in human collaboration. These social interactions are a key component of any human collaboration and must also be considered to achieve the goal of human-like behaviour in robots. Therefore, we must first better understand how humans collaborate with each other and explore the dynamics of human interaction in collaborative tasks so that these insights can later be implemented in robot control systems.

Working in the field of human movement understanding we all face typical questions:

• How does the brain control and coordinate everyday movements?
• What strategies can be used to reconstruct human movements on engineered anthropomorphic systems?
• What advanced computational tools can be used to characterize natural human movements and higher-level strategies?

## 2 The Research Topic on human movement understanding

This Research Topic brings theoretical and experimental insights and outlines guidelines for research activities in the field of human movement understanding. It is intended to provide insights into recent relevant scientific research and to present some examples of the current state of developed technologies that apply these concepts to robotics. The main lines of research can be summarized as.

· Estimation of human motion state by applying robotics: a motion data acquisition protocol for reliable system identification and computational methods based on cluster techniques for dynamic system identification (Sugihara, Kaneta and Murai, 2022); a novel mathematical model of a step- and-brake control of a human subject (Kojima and Sugihara, 2022); and a continuous gait balance model by incorporating knowledge of gait analysis and gait-assist know-how (Yoshikawa, 2022). The research in this cluster provides new research knowledge based on modeling and analysis of balance controllers, and demonstrates the acquired knowledge on humanoid robots.

Estimating human motion using machine learning: overview of database search of published papers from 2012 to mid-2021 that focus on human gait studies and apply machine learning techniques (Harris, Khoo and Demircan, 2022). The research presented in this cluster provides a detailed explanation of human behavior and its application to machine learning, providing valuable expertise for researchers intending to analyze human behavior in the future.

Human-robot interaction: insights into a more natural physical human-robot interaction using human-human motion data (Cabibihan et al., 2022); and analysis and quantification of perturbed human motion during a physical collaborative task (Camilleri, Dogramadzi and Caleb-Solly, 2022). The research presented in this cluster is analyzed in detail, considering the impact on synchrony between close physical interactions between humans and robots.

## 3 Concluding remarks

The developed topics will lead us to answer some questions in the field of human motion understanding. All the works presented in this research topic provide useful know-how and contribute to a better understanding of human motion. Since the field of human movement understanding encompasses numerous scientific disciplines, multidisciplinary research activities, and a wide range of technical and scientific issues, it is not possible to gather all the results that would provide a comprehensive insight into the topic. However, we should all strive to contribute piece by piece to this puzzle in order to expand knowledge to the point where we can no longer distinguish between robotic and human motion.

